# Dendrimers for Drug Delivery

**DOI:** 10.3390/molecules23040938

**Published:** 2018-04-18

**Authors:** Abhay Singh Chauhan

**Affiliations:** School of Pharmacy, Medical College of Wisconsin, 8701 W Watertown Plank Road, Milwaukee, WI 53226, USA; achauhan@mcw.edu; Tel.: +1-414-955-2863

**Keywords:** dendrimer, PAMAM, Tomalia, solubility, stability, transdermal, multifunctional, targeting

## Abstract

Dendrimers have come a long way in the last 25 years since their inception. Originally created as a wonder molecule of chemistry, dendrimer is now in the fourth class of polymers. Dr. Donald Tomalia first published his seminal work on Poly(amidoamine) (PAMAM) dendrimers in 1985. Application of dendrimers as a drug delivery system started in late 1990s. Dendrimers for drug delivery are employed using two approaches: (i) formulation and (ii) nanoconstruct. In the formulation approach, drugs are physically entrapped in a dendrimer using non-covalent interactions, whereas drugs are covalently coupled on dendrimers in the nanoconstruct approach. We have demonstrated the utility of PAMAM dendrimers for enhancing solubility, stability and oral bioavailability of various drugs. Drug entrapment and drug release from dendrimers can be controlled by modifying dendrimer surfaces and generations. PAMAM dendrimers are also shown to increase transdermal permeation and specific drug targeting. Dendrimer platforms can be engineered to attach targeting ligands and imaging molecules to create a nanodevice. Dendrimer nanotechnology, due to its multifunctional ability, has the potential to create next generation nanodevices.

## 1. Introduction

I work in exploring dendrimers as a drug delivery system and mainly use it for entrapment of drugs by non-covalent interactions. This article is a tribute to Dr. Donald Tomalia through my research journey with dendrimers for drug delivery.

While doing my Masters in Pharmacy (M. Pharm), my supervisor asked me to read about dendrimers for my research thesis. I was provided a research paper to read. That was the first time that I saw the dendrimer structure and a picture of a guy with a beard. From that time onwards, dendrimer and Don became synonymous to me.

I also selected dendrimers as my research area for my PhD. My fascination with dendrimers grew as new concepts entered my mind about using Poly(amidoamine) dendrimers (PAMAM) for drug delivery. While working on the dendrimer–indomethacin complex, I ‘discovered’ an inherent anti-inflammatory property of a few PAMAM dendrimers in 2001 (Patent: WO2003080121A1) [1]. I also published the first paper on using PAMAM dendrimers as a transdermal permeation enhancer [2]. Every time I found something interesting on dendrimers, I wanted to share that with Dr. Tomalia.

One of my senior colleagues advised me to send my anti-inflammatory dendrimer work to the International Dendrimer Symposium (IDS-3) in Berlin. Surprisingly, it was selected for a poster presentation. Somehow, as a student, I could arrange funds to travel from India to attend IDS-3 in Berlin. I had never met Don in person and was eagerly waiting for him to come and see my poster, but it did not happen until the end of the day. Then, Dr. Anil Patri visited my poster and brought Don to see my anti-inflammatory dendrimer poster. Don came with a beer mug in his hand. He was very impressed with my findings and, in the next fifteen minutes, he gave me an offer to work with him. I am not sure if it was my research or the beer! About a year later, I ended up joining Dendritic Nanotechnologies in Mount Pleasant, MI, USA.

I am a pharmacist by training and Don exposed me to the vast world of dendrimers and the chemistry behind it. Moreover, it was wonderful to know about the philosophy and history of dendrimers such as the idea that a three-dimensional structured polymer was inspired by a tree and its branches. As a drug delivery scientist, it is imperative to understand the architecture of dendrimers.

## 2. Discussion

### 2.1. Dendrimer-Drug Delivery Strategy

**Figure 1:**
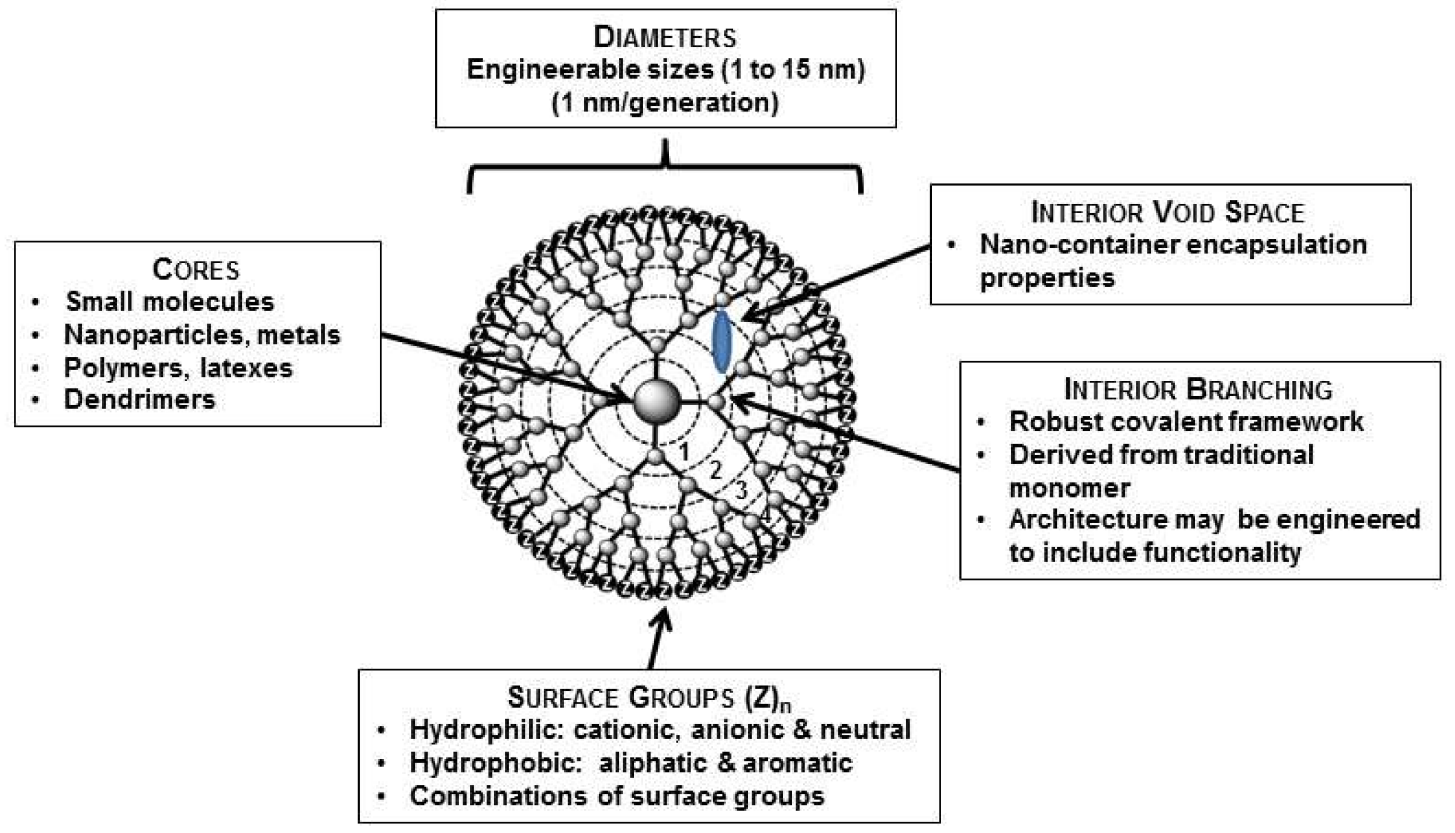
Schematic presentation of a G4 dendrimer containing four generations. Reproduced with permission [8].

PAMAM dendrimers were first reported by Tomalia in 1985 [3,4]. PAMAM dendrimers are one of the most used dendrimers for drug delivery systems [5,6,7]. The dendrimer architecture has three main sites for drug entrapment by using various mechanisms: (i) void spaces (by molecular entrapment); (ii) branching points (by hydrogen bonding); and (iii) outside surface groups by (charge–charge interactions) (Figure 1).

**Scheme 1:**
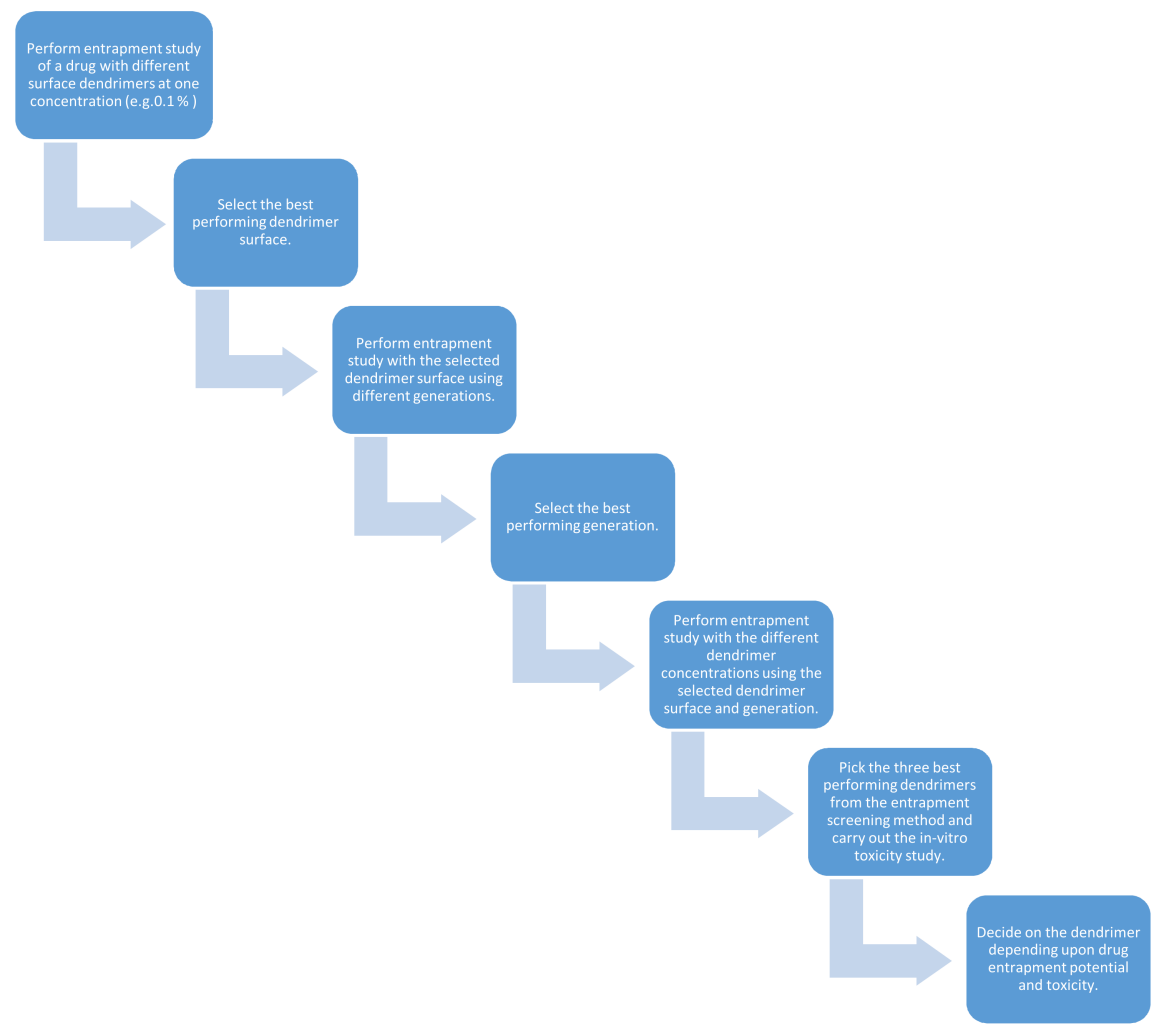
Strategy to select dendrimers for drug entrapment.

Entrapment of a drug depends on the structures of both the dendrimer and the drug. Hence, it is important to understand the chemistry behind the dendrimer architecture. Designing or selecting an appropriate dendrimer is the main step for drug entrapment. As of now, we have no tools to predict the entrapment potential of a dendrimer for any drug. Hence, screening with different dendrimers is suggested to find the right combinations. PAMAM dendrimers are commercially available with amine, hydroxyl, carboxylate and pyrrolidinone surfaces along with corresponding generations. Here is a suggested strategy to find the appropriate dendrimer for a drug molecule (Scheme 1).

### 2.2. Multifunctional Delivery System

A dendrimer can perform various jobs ranging from solubility enhancement to drug targeting. We envisaged that dendrimers would have the potential to be used as multifunctional excipients. My current research focus is to explore dendrimers for multifunctional capability by enhancing solubility, dissolution, gastrointestinal tract (GIT) permeability, bioavailability, stability, multiple drug entrapment, controlled delivery, and efficacy of drugs in a single formulation. Various interrelated approaches are investigated simultaneously to establish dendrimers as a multifunctional delivery system. Here is the classification of various categories and sub-categories to define the role of dendrimer:

- Pre-formulation: solubility, stability, storage and transport,
- Formulation: dissolution, stability, enzymatic and hydrolytic degradation, permeability and multiple drug entrapment,
- Advanced formulation: bioavailability, targeting, toxicity, transdermal, novel drug delivery.

a. *Solubility enhancers*: Some of the newly developed and even launched drugs are rejected by the pharmaceutical industry and/or exhibit sub-optimal performance because of low water solubility and hence low bioavailability. We have published one of the earlier works on aqueous solubility enhancement propensity of dendrimers [2,9,10].

**Figure 2:**
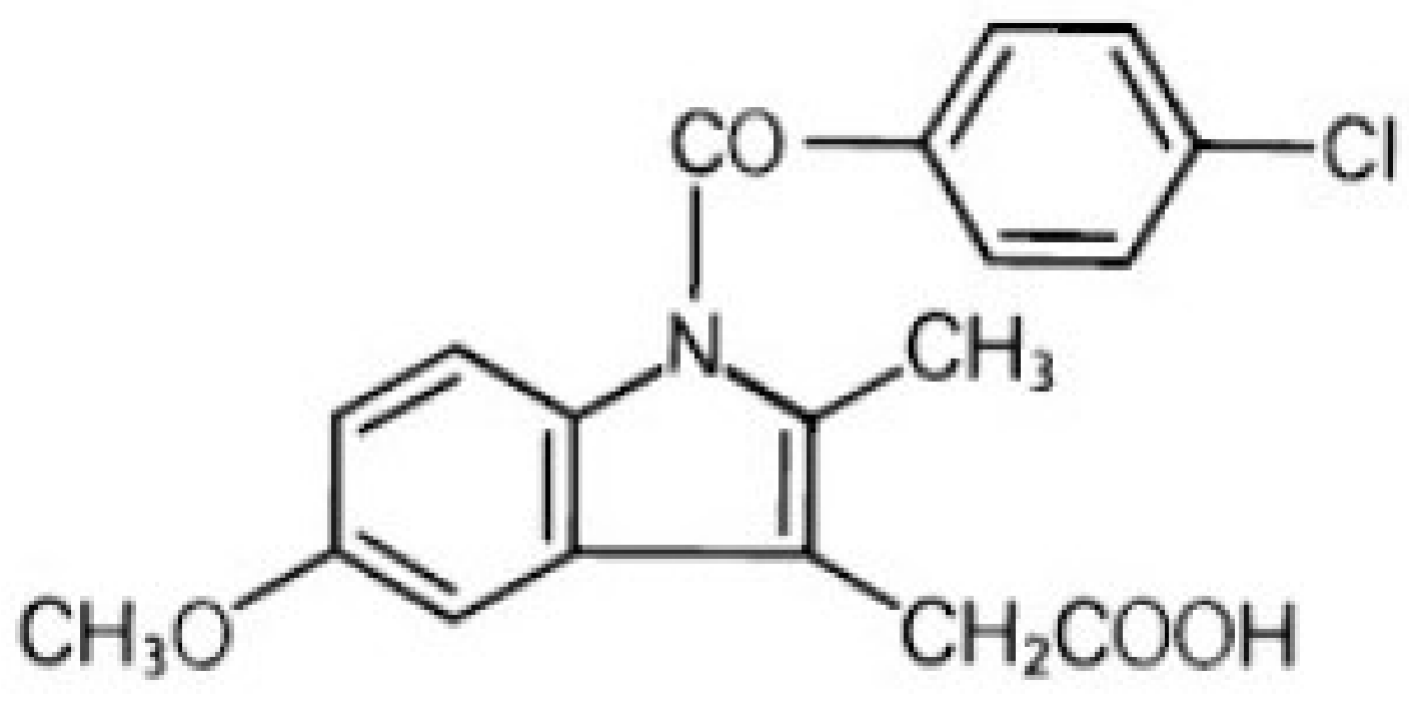
Structure of indomethacin.

PAMAM dendrimers are water-soluble molecules and their ability to entrap hydrophobic molecules makes them good solubility enhancers [11]. Hydrophobic molecules can be entrapped in dendrimers using mechanisms discussed before (Scheme 1). Indomethacin (Figure 2) is a weakly acidic drug and it was entrapped in different dendrimers. It showed the best solubility enhancement with G4-NH2 and low performance with G4-COOH dendrimer. The carboxylate groups of indomethacin were associated with surface amine groups on G4-NH2 dendrimers through charge–charge interactions, which is not feasible with G4-COOH dendrimers. Nonetheless, some entrapment with G4-COOH dendrimers was observed due to the molecular encapsulation.

**Figure 3:**
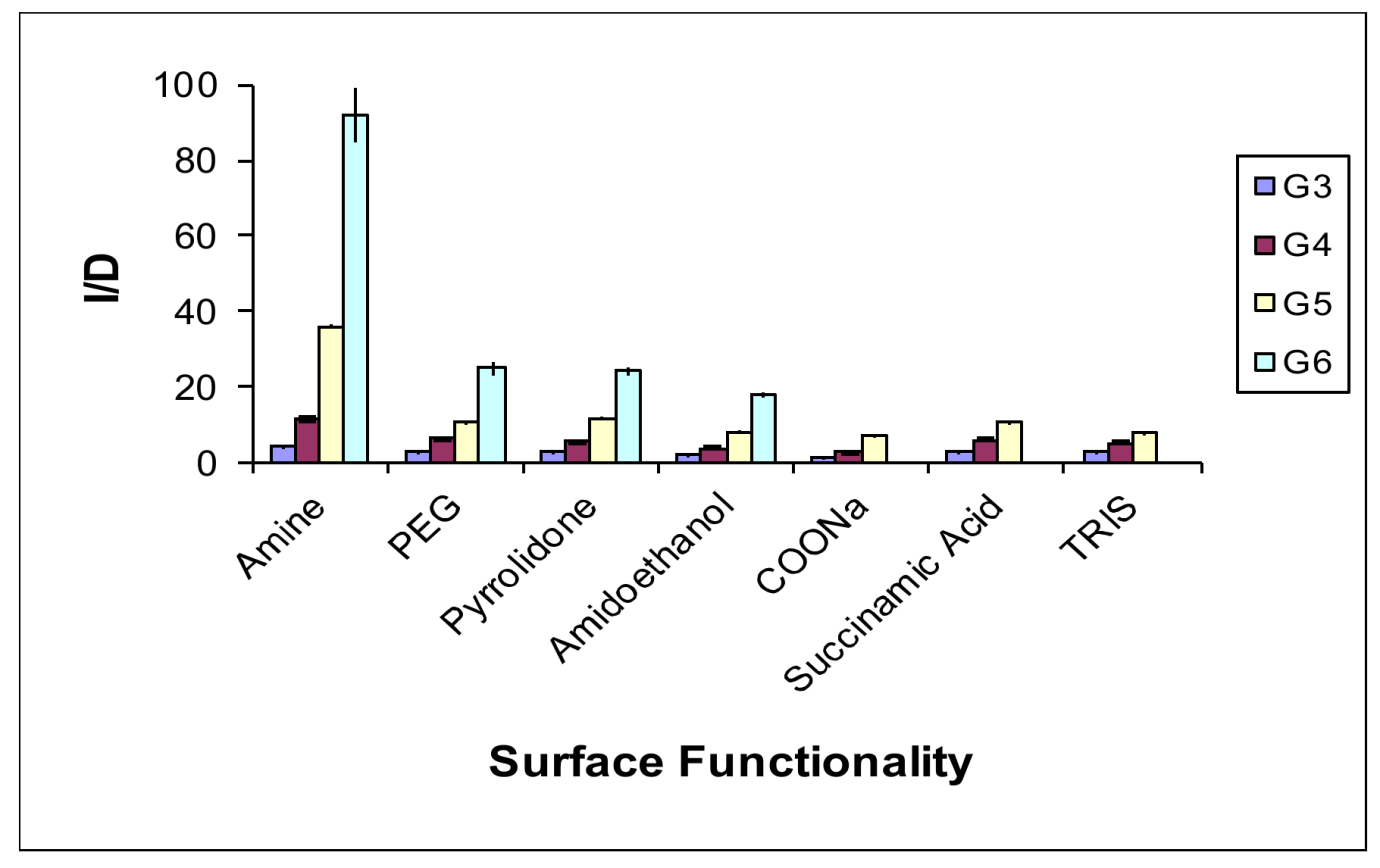
Effect of dendrimer surface and generation on entrapment potential (I/D) of dendrimers. Reproduced with permission [8].

Drug entrapment was also size dependent and increased with the increase in dendrimer generations (Figure 3). As charge–charge interactions represent one of the main mechanisms in drug entrapment, pH plays a vital role. We have shown that drug entrapment occurs best in a pH, where both dendrimer and drug are fully ionized [10]. It means that selecting an appropriate pH for entrapment is very crucial.

**Figure 4:**
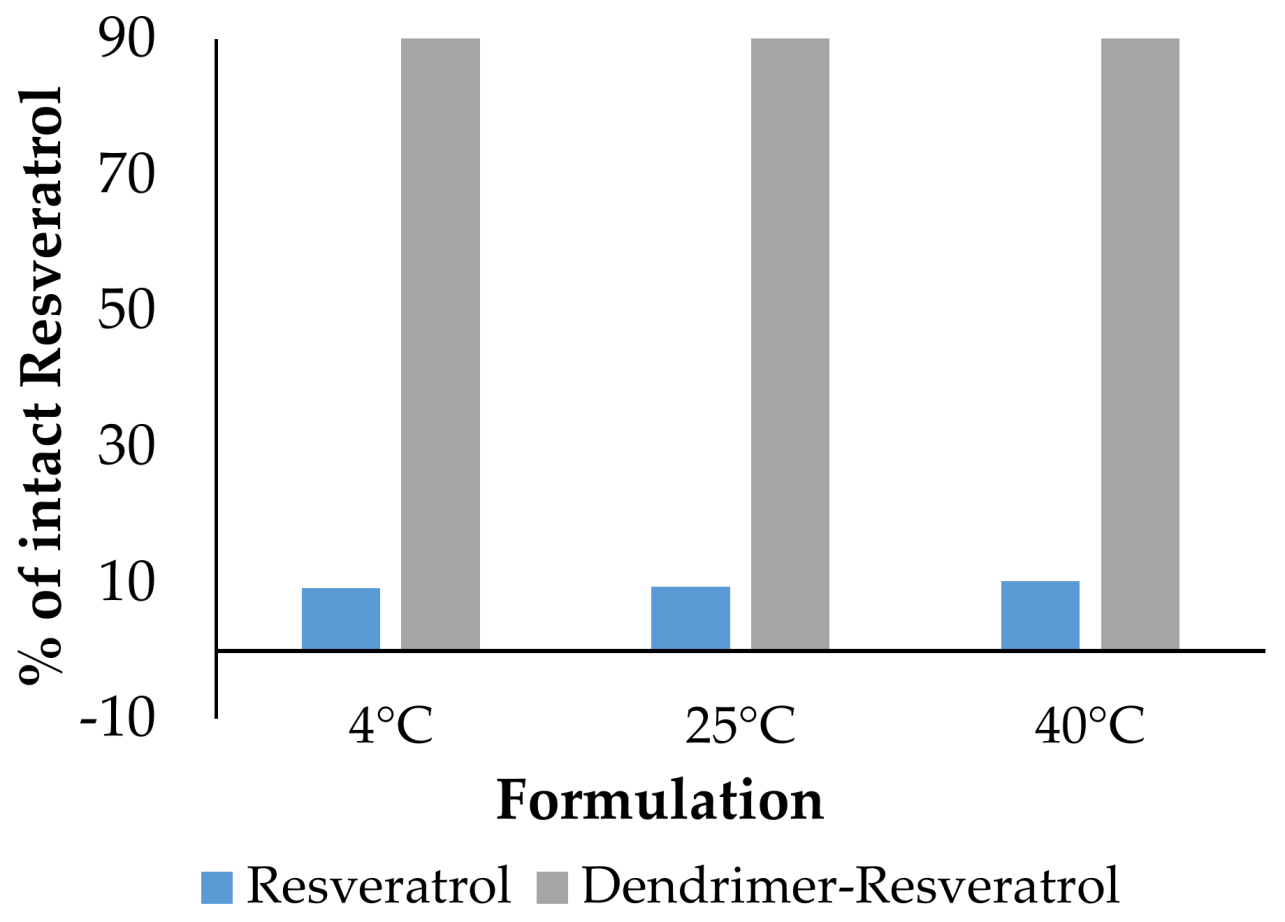
Percentage of intact resveratrol remaining in the formulations after Day 1.

**Figure 5:**
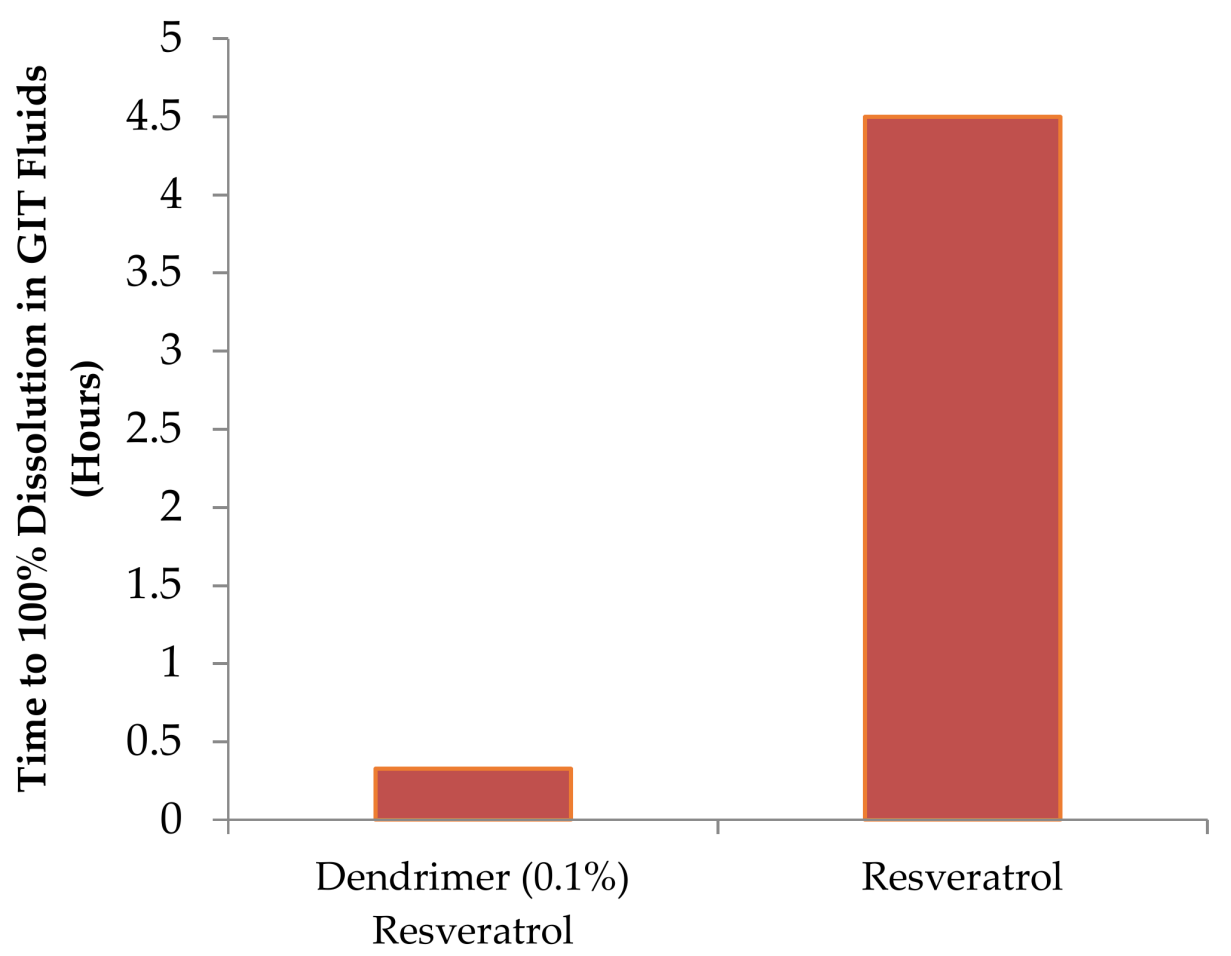
Dissolution profile of dendrimer–resveratrol formulations.

Once dendrimer entraps/solubilizes a hydrophobic molecule, this dendrimer-drug complex can also be used to enhance drug dissolution, stability and bioavailability.

b. *Stability:* Dendrimers provide stability to the guest molecules in vitro and in vivo. Resveratrol is a hydrophobic molecule with stability issues. The entrapment of resveratrol in dendrimer enhanced its water solubility and stability (Figure 4) [12].

c. *Dissolution*: Dendrimer-drug complexes showed very high and fast drug dissolution compared to the hydrophobic drug alone (Figure 5) [12].

d. *Drug Release*: Release of drugs from dendrimers can be controlled by various means.

(i) Chemical modification of dendrimer: Indomethacin showed slow release with G4-NH2 dendrimer while fast release was observed with G4-COOH [13]. The slow release with G4-NH2 dendrimer can be attributed to the strong interactions of drugs with dendrimers.
(ii) Physical loading: Rate of drug release from dendrimers can also be modified by adjusting dendrimer-to-drug molar ratio. We have shown that, by changing cisplatin loading in PAMAM dendrimers, drug release can be controlled [14]. Cisplatin release from dendrimers showed fast release with higher cisplatin/dendrimer molar ratio formulation, whereas lowering the cisplatin/dendrimer molar ratio would provide slower release of cisplatin.

e. *Oral Bioavailability*: Entrapment of drugs in dendrimers helps to enhance solubility, stability and dissolution. These properties eventually help to enhance oral bioavailability of the drug. Simvastatin (SMV) was entrapped in PAMAM dendrimers to prepare a dendrimer–simvastatin (SMV) complex [15]. Dendrimer–SMV formulations showed better oral bioavailability than pure SMV suspension following oral administration [16]. Peak plasma SMV concentration increased from 2.3 μg/mL with pure SMV to 3.8 μg/mL with dendrimer formulations. The mean SMV residence time was 3–5 times better for the dendrimer–SMV formulation. 

f. *Multiple drug delivery systems*: PAMAM dendrimers were evaluated to explore the potential of PAMAM dendrimers for multiple drug delivery. We have entrapped two anti-hypertensive drugs Ramipril (RAPL) and hydrochlorothiazide (HCTZ [17]). The results showed that the solubility of RAPL and HCTZ was dependent on dendrimer concentrations and pH of dendrimer solution. Formulations of dendrimer-drug complex and dendrimer-drug combinations (RAPL+HCTZ) displayed faster dissolution in simulated gastric fluid at pH 1.2 and United States Pharmacopeia (USP) dissolution medium at pH 7.0. We have demonstrated dendrimer’s capability for combination therapy with predictable dissolution profile by formulating RAPL and HCTZ for the treatment of hypertension.

### 2.3. Dendrimer-Mediated Transdermal Delivery 

**Figure 6:**
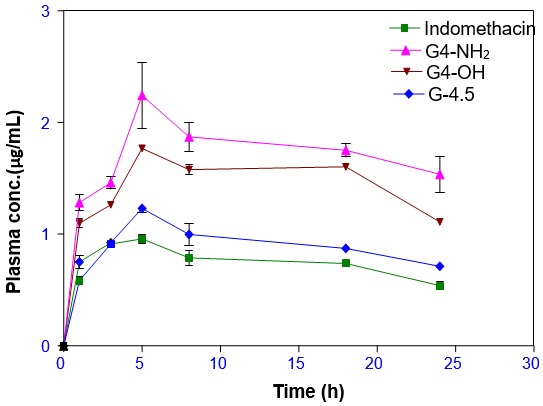
Transdermal delivery of dendrimer formulations (G4-NH2, G4-OH, G4.5-COOH dendrimers, indomethacin). Reproduced with permission [2].

The transdermal delivery of indomethacin formulations with different dendrimers was studied and transdermal permeation potential was evaluated (Figure 6) [2]. The effect of concentrations of dendrimers was observed and it showed a linear increase in flux with an increasing concentration of each of the dendrimers. Transdermal permeation results showed contrasting behavior compared to the solubility studies, where Higuchi’s A**_N_** solubility profile was noted [10]. The steady-state flux of the dendrimer-drug formulations showed 2–4.5 times enhancement factor compared to the pure drug formulation. Entrapment of resveratrol in dendrimer increases its solubility and stability and the dendrimer–resveratrol complex showed significantly higher (2.5 times) transdermal permeation compared to resveratrol alone.

### 2.4. Engineered Dendrimers 

**Figure 7:**
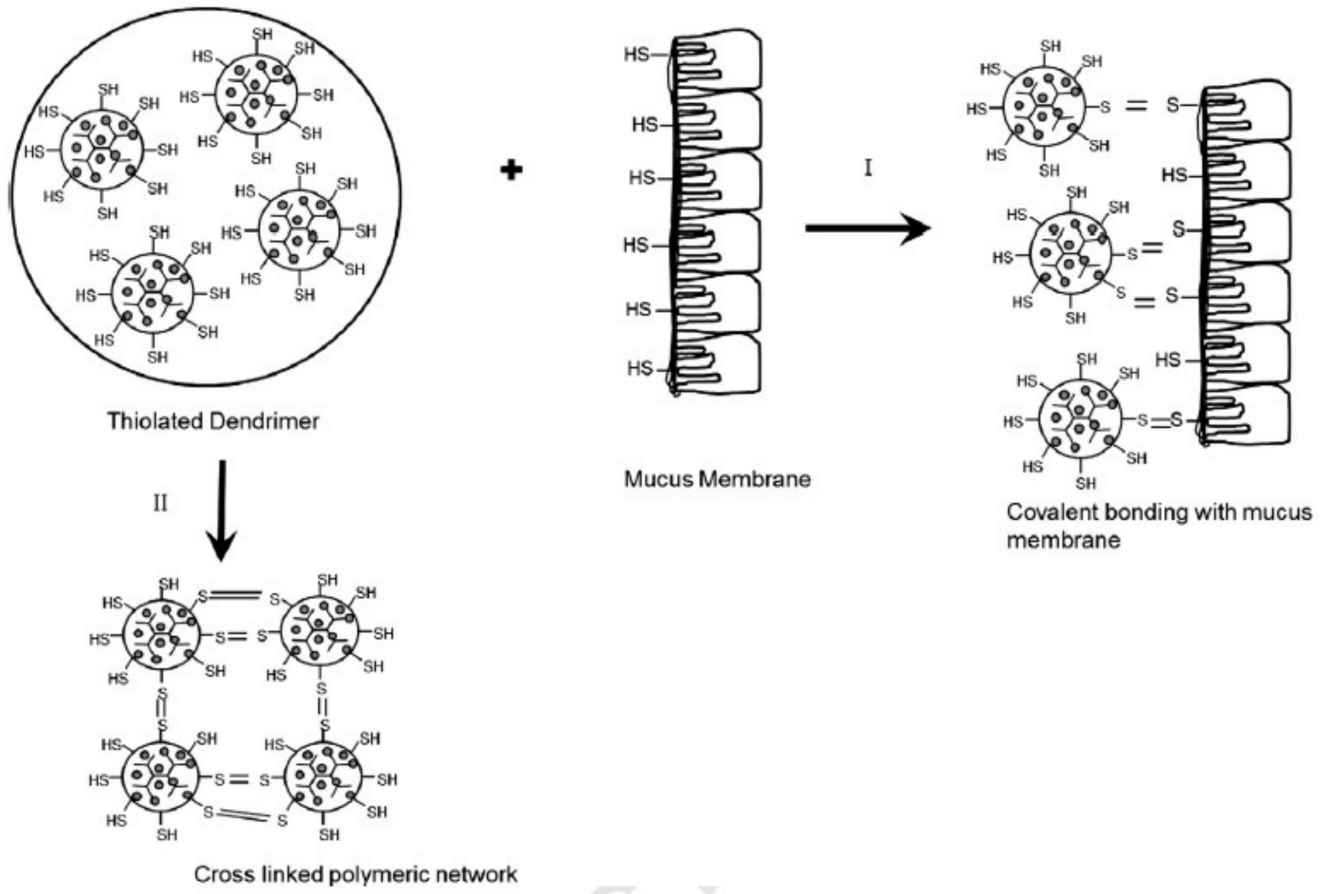
Schematic representation of muco-adhesiveness of thiolated dendrimers. (**I**) disulfide bond formation with glycoproteins of mucus membrane; (**II**) inter cross linking of thiolated dendrimers to form polymeric network. Reproduced with permission [19].

Dendrimer platform can be engineered to develop various applications. We have developed thiolated dendrimers for enhanced mucoadhesion [18,19]. The thiol groups on the polymers form covalent bonds with the cysteine domains of the mucin to achieve mucoadhesion. Dendrimers were decorated with the thiol groups using its polyvalent architecture (Figure 7). Thiolated dendrimers were loaded with acyclovir and the dendrimer-drug complexes have shown encouraging results for drug loading, bio adhesion, and controlled release profile.

### 2.5. Hybrid Dendrimers

**Figure 8:**
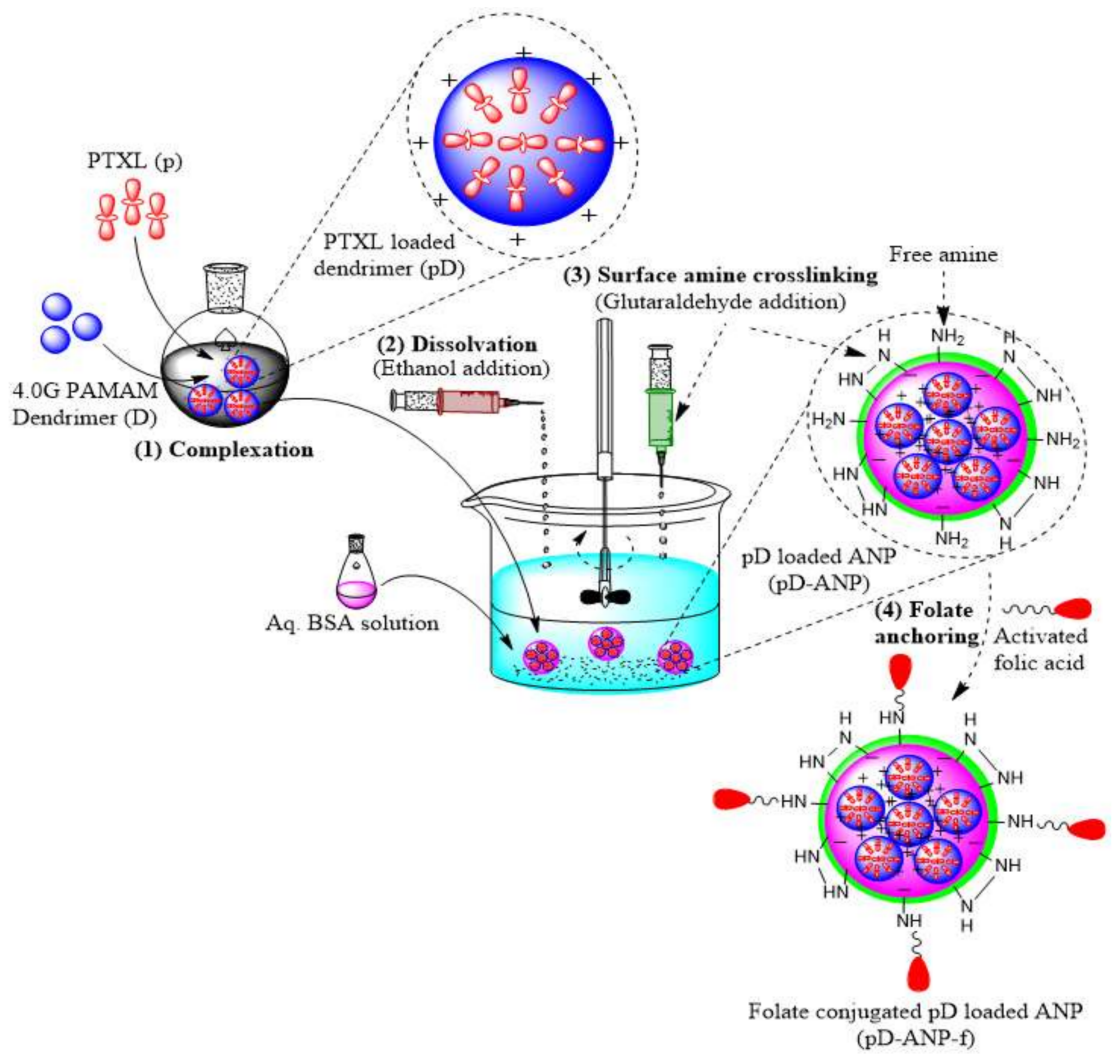
PAMAM dendrimer-Albumin nanoparticle nano-assembly for delivery of paclitaxel. Reproduced with permission [20].

1. Dendrimer-nanoparticle nano-assembly: Dendrimers can be assembled with other delivery systems to create a novel nanostructure assembly. We have prepared a self-assembled nanostructure assembly by controlled electrostatic gelation of anionic albumin with 4.0 G PAMAM dendrimers [20]. Dendrimers were mainly utilized for loading of paclitaxel, and albumin nanoparticles were used to anchor folate as a targeting ligand (Figure 8). This dendrimer–nanoparticle assembly provides a novel way of targeting drugs to the cancer cells compared to paclitaxel and albumin nanoparticles.

2. Dendrimer-Dendrimer nano-assembly: We have demonstrated that PAMAM dendrimers with different surfaces can be assembled together to create hybrid dendrimers [18]. Each dendrimer portrays a characteristic release profile for a drug. For example, dendrimers with amine surface groups showed slow release and dendrimers with carboxylate surface depict fast release of indomethacin. Hence, two or more dendrimers can be combined to work in tandem in a formulation, thereby achieving a therapeutically desired release profile.

## 3. Conclusions

We have demonstrated that PAMAM dendrimers can enhance aqueous solubility, stability, dissolution, drug release, targeting and pharmacokinetics of various drugs. The future of drug delivery would be fabricating ‘nano-robots’ capable of doing multiple jobs inside the body. These ‘nano-robots’ can either be biodegradable or leave the body through excretion routes. Designing a sophisticated drug delivery platform needs an interdisciplinary approach involving chemistry, engineering and pharmacy disciplines. Dendrimer nanotechnology due to its multifunctional ability has the potential to create next generation drug delivery platforms. The future of dendrimers would be to exploit their multifunctional capabilities and engineerable platforms. The idea that sprouted through the ‘tree branches’ has now taken ‘root’ in drug delivery.

## References

[1] Chauhan A.S., Diwan P.V., Jain N.K., Tomalia D.A. (2009). Unexpected in vivo anti-inflammatory activity observed for simple, surface functionalized poly(amidoamine) dendrimers. Biomacromolecules.

[2] Chauhan A.S., Sridevi S., Chalasani K.B., Jain A.K., Jain S.K., Jain N.K., Diwan P.V. (2003). Dendrimer-mediated transdermal delivery: Enhanced bioavailability of indomethacin. J. Control. Release.

[3] Tomalia D., Baker H., Dewald J., Hall M., Kallos G., Martin S., Roeck J., Ryder J., Smith P. (1985). A New Class of Polymers: STARBURST®-Dendritic Macromolecules. Polym. J..

[4] Tomalia D., Naylor A.M., Goddard W.A. (1990). STARBURST Dendrimers: Molecular Level Control of Size, Shape, Surface Chemistry, Topology and Flexibility from Atoms to Macroscopic Matter. Angew. Chem. Int. Ed. Engl..

[5] Tomalia D.A. (1991). Dendrimer research. Science.

[6] Tomalia D.A. (2009). In quest of a systematic framework for unifying and defining nanoscience. J. Nanopart. Res..

[7] Tomalia D.A. (2012). Interview: An architectural journey: From trees, dendrons/dendrimers to nanomedicine. Interview by Hannah Stanwix. Nanomedicine.

[8] Chauhan A.S. (2015). Dendrimer nanotechnology for enhanced formulation and controlled delivery of resveratrol. Ann. N. Y. Acad. Sci..

[9] Asthana A., Chauhan A.S., Diwan P.V., Jain N.K. (2005). Poly(amidoamine) (PAMAM) dendritic nanostructures for controlled site-specific delivery of acidic anti-inflammatory active ingredient. AAPS PharmSciTech.

[10] Chauhan A.S., Jain N.K., Diwan P.V., Khopade A.J. (2004). Solubility enhancement of indomethacin with poly(amidoamine) dendrimers and targeting to inflammatory regions of arthritic rats. J. Drug Target..

[11] Svenson S., Chauhan A.S. (2008). Dendrimers for enhanced drug solubilization. Nanomedicine.

[12] Chauhan A., Newenhouse E., Gerhardt A. (2018). Compositions Comprising a Dendrimer-Resveratrol Complex and Methods for Making and Using the Same. U.S. Patent.

[13] Chauhan A., Svenson S., Reyna L., Tomalia D. (2007). Solubility enhancement propensity of PAMAM nanoconstructs. Mater. Matters Nanomater..

[14] Kulhari H., Pooja D., Singh M.K., Chauhan A.S. (2015). Optimization of carboxylate-terminated poly(amidoamine) dendrimer-mediated cisplatin formulation. Drug Dev. Ind. Pharm..

[15] Kulhari H., Pooja D., Prajapati S.K., Chauhan A.S. (2011). Performance evaluation of PAMAM dendrimer based simvastatin formulations. Int. J. Pharm..

[16] Kulhari H., Kulhari D.P., Prajapati S.K., Chauhan A.S. (2013). Pharmacokinetic and pharmacodynamic studies of poly(amidoamine) dendrimer based simvastatin oral formulations for the treatment of hypercholesterolemia. Mol. Pharm..

[17] Singh M.K., Pooja D., Kulhari H., Jain S.K., Sistla R., Chauhan A.S. (2017). Poly(amidoamine) dendrimer-mediated hybrid formulation for combination therapy of ramipril and hydrochlorothiazide. Eur. J. Pharm. Sci..

[18] Chauhan A., Svenson S. (2007). Formulations Containing Hybrid Dendrimers.

[19] Yandrapu S.K., Kanujia P., Chalasani K.B., Mangamoori L., Kolapalli R.V., Chauhan A. (2013). Development and optimization of thiolated dendrimer as a viable mucoadhesive excipient for the controlled drug delivery: An acyclovir model formulation. Nanomedicine.

[20] Tekade R.K., Tekade M., Kumar M., Chauhan A.S. (2015). Dendrimer-stabilized smart-nanoparticle (DSSN) platform for targeted delivery of hydrophobic antitumor therapeutics. Pharm. Res..

